# Chronic periodontal disease is related with newly developing hypertension: a nationwide cohort study

**DOI:** 10.1186/s40885-024-00285-6

**Published:** 2024-10-01

**Authors:** Jung-Hyun Park, Min Kyoung Kang, Gwang Hyun Leem, Jin-Woo Kim, Tae-Jin Song

**Affiliations:** 1 Department of Oral and Maxillofacial Surgery, Mokdong Hospital, Ewha Womans University College of Medicine, Seoul, Republic of Korea; 2 Department of Neurology, Seoul Hospital, Ewha Womans University College of Medicine, 260, Gonghang-daero, Gangseo-gu, Seoul, 07804 Republic of Korea; 3 Convergence Medicine, Seoul Hospital, Ewha Womans University College of Medicine, Seoul, Republic of Korea

**Keywords:** Periodontal disease, Periodontitis, Hypertension, Epidemiology

## Abstract

**Background:**

Periodontal disease (PD) is a condition that can be treated and managed. This study aimed to determine if chronic PD status is associated with the risk of developing hypertension, utilizing data from the National Health Insurance Database of Korea.

**Methods:**

Participants who received oral health examinations both in 2003 and in 2005–2006 were included. Those with a history of hypertension were excluded. Hypertension was defined as at least one outpatient or inpatient claim diagnosis (primary or secondary) of hypertension (International Classification of Diseases (ICD)-10 codes I10-I11) with prescription for antihypertensive medication or at least one incident of systolic blood pressure greater than 140 mmHg or diastolic blood pressure greater than 90 mmHg during a health examination. Changes of PD status was determined during two oral examinations. Study participants were divided into 4 groups according to the changes of PD status: PD-free (those consistently free of disease in both exams), PD-recovered (individuals with disease initially but not in the second exam), PD-developed (no disease initially, but present in the second exam), and PD-chronic (disease throughout both exams). The incidence of hypertension after the second oral health examination (index date) was monitored. Participants were observed from the index date until the earliest occurrence of hypertension onset, mortality, or December 2020.

**Results:**

The study comprised 706,584 participants: 253,003(35.8%) in the PD-free group, 140,143(19.8%) in the PD-recovered group, 132,397(18.7%) in the PD-developed group, and 181,041(25.6%) in the PD-chronic group. Over a median follow-up duration of 14.3 years, 239,937 (34.0%) cases of hypertension were recorded. The PD-recovered group had a lower risk of hypertension compared to the PD-chronic group, while the PD-developed group had a higher risk of hypertension compared to the PD-free group.

**Conclusion:**

Chronic PD is associated with an increased risk of developing hypertension. Although the increase in risk is modest, recovery from PD may have beneficial effects in reducing hypertension risk. Further studies are needed to confirm the importance of regular dental examinations and effective management of PD to reduce hypertension risk.

**Supplementary Information:**

The online version contains supplementary material available at 10.1186/s40885-024-00285-6.

## Background

Hypertension is a significant global health issue; estimates suggest that approximately 1.4 billion individuals worldwide will be affected by this condition by 2025 [1]. Due to its strong association with cardiovascular diseases, stroke, and mortality, hypertension preventive strategies are essential [2, 3]. Hypertension is highly influenced by various environmental factors and genetic predisposition. Modifiable lifestyle factors, such as high salt intake, inadequate potassium intake, weight gain, and lack of exercise can increase the risk of hypertension. However, even when these factors are addressed, some hypertension risk remains. Therefore, further research is necessary to identify additional associated and risk factors that may be modified for prevention of hypertension [4].

Periodontal disease (PD) is widespread among the general population. This condition constitutes a gingival infection that harms the periodontal tissues. If not addressed, it can result in the deterioration of the underlying bone structure that provides support to the teeth. Moreover, PD can induce transient bacteremia and systemic inflammation; these play a role in systemic diseases such as hypertension [5–7]. Therefore, PD has the potential to be a notable contributing factor to the development of hypertension. PD is a dynamic condition that can vary over time based on factors such as oral hygiene practices, access to dental care, and individual health behaviors. While static PD status at a single point in time provides useful information, it does not capture the full extent of the disease’s impact on systemic health. The alterations in PD status provide information about the disease’s progression or improvement, reflecting changes in the inflammatory burden and bacterial load in the body. These changes may influence the risk of developing hypertension and could be targeted for prevention strategies.

However, to date, no large-scale study has examined the association between PD persistency or improvement and the risk of hypertension. We postulated that the risk of developing hypertension may differ depending on the persistence of or recovery from PD. Our study aimed to investigate the correlation between alterations in PD status and the risk of hypertension in a large-scale longitudinal study of a national population.

## Methods

### Database

This study utilized data from the Korean National Health Insurance Service (NHIS). The NHIS provides a nation-supported health examination and medical care institution database for research purposes [8]. The NHIS is the primary healthcare insurer in Korea, providing coverage to approximately 97% of the population; the remaining 3% receive assistance through the Medical Aid program [9, 10]. NHIS subscribers are encouraged to undergo medical health screenings every one to two years [11, 12]. The database contains demographic information; medical claims data; and health examination information including height, weight, household income, lifestyle associated with health, and oral health status as determined by dental examination [13–15]. This study was approved by the Institutional Review Board of Ewha Womans University College of Medicine (approval number 2021–07–034), and the need for consent was waived.

### Study population and variables

**Fig. 1:**
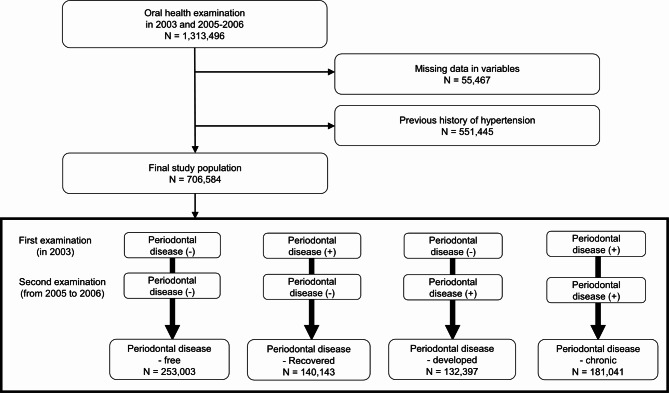
Flow chart of study participant selection

We obtained data from the NHIS concerning individuals aged 20 and above who underwent an oral health examination in 2003. NHIS granted access to a database of 2,415,963 individuals (dataset number: NIHS-2022-01-313). For our analysis, we included all participants (*n* = 1,313,496) who received oral health exams both in 2003 and either 2005 or 2006. Individuals with incomplete data for the variables of interest – age, sex, body mass index, household income, smoking status, alcohol consumption, regular physical activity, and the results of the oral examination – were excluded (*n* = 55,467). Those with a history of hypertension (*n* = 551,445) were also excluded. Hypertension was defined as at least one outpatient or inpatient claim diagnosis (primary or secondary) of hypertension (International Classification of Diseases (ICD)-10 codes I10-I11) with prescription for antihypertensive medication, or at least one incident of systolic blood pressure (SBP) greater than 140 mmHg or diastolic blood pressure (DBP) greater than 90 mmHg during a health examination. Given that hypertension awareness and treatment rates are 74.1% and 70.3% respectively, as reported by the Korea Hypertension Fact Sheet 2023 [16], using claims data might lead to under-reporting bias. To mitigate this, we included both claim data and health examination results to define hypertension, aiming to improve the accuracy of hypertension identification. The study included a total of 706,584 participants (Fig. 1).

Dentists who had undergone training prior to the examinations conducted the oral assessments including PD and number of tooth loss. The assessment of PD involved measuring periodontal pocket depth, a widely accepted indicator of periodontal tissue damage, as well as evaluating gingival inflammation [17, 18]. Periodontitis was defined as the presence of at least one pocket with a probing depth of 4 mm or more, taking into consideration the absence of pockets deeper than 3 mm in a healthy periodontium. Gingival inflammation was identified by signs such as redness, swelling, a shiny appearance, or bleeding upon probing. Participants were divided into four groups according to changes in their PD status over the two oral exams. The groups consisted of: (1) PD-free (those consistently free of disease in both exams), (2) PD-recovered (individuals with disease initially but not in the second exam), (3) PD-developed (no disease initially, but present in the second exam), and (4) PD-chronic (disease throughout both exams) [17].

The incidence of hypertension was the primary outcome. Participants were followed from the second oral health examination in 2005–2006, which served as the index date, until December 2020. Follow-up was censored in case of hypertension onset, mortality, or by December 2020. As per the hypertension management guidelines, optimal blood pressure is defined as SBP values below 120 mmHg and/or DBP values below 80 mmHg [19, 20]. Hypertension was defined as at least one outpatient or inpatient claim diagnosis (primary or secondary) of hypertension (ICD-10 codes I10-I11) with prescription for antihypertensive medication or at least one incident of SBP greater than 140 mmHg or DBP greater than 90 mmHg during a health examination after the index date.

As for covariates, the information recorded on the index date encompassed age, sex, body mass index, and household income. Information about smoking patterns, alcohol consumption (frequency per week), and physical activity (frequency per week) was gathered through self-administered surveys. Smoking status was categorized into non-smoker, former-smoker, and current smoker. The number of tooth loss was assessed by a dentist upon oral health examination, categorized as 0, 1–7, 8–14, and ≥ 15 without considering the cause. Further explanations regarding comorbidities and the Charlson Comorbidity Index are detailed in the Supplementary Methods Sects. [21–31].

### Statistical analysis

For baseline characteristic assessment, categorical variables underwent Chi-square testing to compare between groups. Continuous variables were examined through analysis of variance with subsequent Bonferroni post-hoc analysis. The connection between changes in PD status and hypertension incidence was explored using Kaplan-Meier survival curves, followed by log-rank test evaluation for significance. To analyze hazard ratio (HR), Cox proportional hazard regression analysis was performed, adjusting for potential confounders. Multivariable Cox regression incorporated adjustments for variables including age, sex, body mass index, household income, smoking habits, alcohol consumption, physical activity, comorbidities (diabetes mellitus, dyslipidemia, atrial fibrillation, cancer, renal disease), and Charlson Comorbidity Index. HR and 95% confidence interval (CI) represented the results of Cox regression analysis. Schoenfeld residuals were used to assess the proportional hazards assumption, and no violations were detected. Through pairwise comparison analysis, we assessed the alterations in hypertension risk among individuals who experienced PD recovery or developed new-onset PD (PD-recovered group vs. PD-chronic group and PD-developed group vs. PD-free group). Furthermore, we examined the disparities in hypertension risk based on the history of PD (PD-recovered group vs. PD-free group and PD-developed group vs. PD-chronic group). In order to minimize the potential for reverse causality, a sensitivity analysis was conducted by excluding participants with a diagnosis of hypertension within one year from the index date (landmark analysis). Statistical analysis was conducted using SAS software (version 9.2, SAS Institute, Cary, NC). Statistical significance was defined as *p*-values < 0.05.

## Results

All participants, amounting to 253,003 (35.8%), 140,143 (19.8%), 132,397 (18.7%), and 181,041 (25.6%), were categorized into the PD-free, PD-recovered, PD-developed, and PD-chronic groups, respectively. Table 1 presents baseline characteristics upon changes in PD status. Participants’ average age was 39.55 ± 10.11 years, with 65.9% being male. The PD-chronic group comprised a larger proportion of males in contrast to the other groups, while the PD-free group had a greater share of females. The PD-chronic group demonstrated the highest frequency of current smoking and alcohol consumption, the PD-developed and PD-recovered groups exhibited lower frequencies, and the PD-free group demonstrated the lowest. Regarding associated conditions, diabetes mellitus, dyslipidemia, and renal disease exhibited lower prevalence in the PD-free group, but were more prevalent in the PD-recovered and PD-developed groups. The highest prevalence was seen in the PD-chronic group. Conversely, the frequency of cancer and a Charlson Comorbidity Index ≥ 2 was greatest in the PD-free group when compared to the remaining groups (Table 1).

**Table 1:** Baseline characteristics of the study population

	Total	Periodontal disease-free	Periodontal disease -recovered	Periodontal disease -developed	Periodontal disease -chronic	*p*-value
**No. of patients**	709,584	253,003 (35.8)	140,143 (19.8)	132,397 (18.7)	181,041 (25.6)	
**Age**,** years**	39.55 ± 10.11	38.57 ± 10.23	39.53 ± 10.15	39.7 ± 9.88	40.84 ± 9.95	< 0.001
**Sex**						< 0.001
Men	465,780 (65.9)	145,856 (57.7)	92,930 (66.3)	90,825 (68.6)	136,169 (75.2)	
Women	240,804 (34.1)	107,147 (42.4)	47,213 (33.7)	41,572 (31.4)	44,872 (24.8)	
**BMI (kg/m** ^**2**^ **)**	22.87 ± 3.56	22.58 ± 2.82	22.91 ± 2.87	22.98 ± 5.7	23.17 ± 2.86	< 0.001
**Household income**						< 0.001
Q1, lowest	83,594 (11.8)	30,295 (12.0)	16,528 (11.8)	15,110 (11.4)	21,661 (12.0)	
Q2	267,349 (37.8)	97,529 (38.6)	53,421 (38.1)	47,882 (36.2)	68,517 (37.9)	
Q3	247,370 (35.0)	87,722 (34.7)	48,910 (34.9)	47,706 (36.0)	63,032 (34.8)	
Q4, highest	108,271 (15.3)	37,457 (14.8)	21,284 (15.2)	21,699 (16.4)	27,831 (15.4)	
**Smoking status**						< 0.001
None	406,371 (57.5)	166,502 (65.8)	80,889 (57.7)	73,106 (55.2)	85,874 (47.4)	
Former	87,383 (12.4)	30,983 (12.3)	17,552 (12.5)	16,876 (12.8)	21,972 (12.1)	
Current	212,830 (30.1)	55,518 (21.9)	41,702 (29.8)	42,415 (32.0)	73,195 (40.4)	
**Alcohol consumption** **(days/week)**						< 0.001
< 1	2125 (0.3)	592 (0.2)	443 (0.3)	409 (0.3)	681 (0.4)	
1–2	489,194 (69.2)	185,724 (73.4)	97,116 (69.3)	89,974 (68.0)	116,380 (64.3)	
≥ 3	215,265 (30.5)	66,687 (26.4)	42,584 (30.4)	42,014 (31.7)	63,980 (35.3)	
**Regular physical activity** **(days/week)**						< 0.001
< 3	585,338 (82.8)	208,546 (82.4)	115,765 (82.6)	109,771 (82.9)	151,256 (83.6)	
≥ 3	121,246 (17.2)	44,457 (17.6)	24,378 (17.4)	22,626 (17.1)	29,785 (16.5)	
**Comorbidities**						
Diabetes mellitus	59,994 (8.5)	20,072 (7.9)	11,444 (8.2)	11,526 (8.7)	16,952 (9.4)	< 0.001
Dyslipidemia	117,140 (16.6)	39,639 (15.7)	23,722 (16.9)	22,027 (16.6)	31,752 (17.5)	< 0.001
Atrial fibrillation	643 (0.1)	251 (0.1)	122 (0.1)	122 (0.1)	148 (0.1)	0.298
Cancer	9542 (1.4)	3687 (1.5)	1891 (1.4)	1777 (1.3)	2187 (1.2)	< 0.001
Renal disease	1770 (0.3)	601 (0.2)	355 (0.3)	331 (0.3)	483 (0.3)	0.300
**Charlson Comorbidity Index**						< 0.001
0	318,452 (45.1)	111,209 (44.0)	63,061 (45.0)	59,423 (44.9)	84,759 (46.8)	
1	293,793 (41.6)	107,743 (42.6)	58,245 (41.6)	55,359 (41.8)	72,446 (40.0)	
≥ 2	94,339 (13.4)	34,051 (13.5)	18,837 (13.4)	17,615 (13.3)	23,836 (13.2)	
**Number of tooth loss**						< 0.001
0	608,792 (86.2)	224,383 (88.7)	121,533 (86.7)	113,078 (85.4)	149,978 (82.8)	
1–7	93,692 (13.3)	27,278 (10.8)	17,905 (12.5)	18,553 (14.0)	29,956 (16.6)	
8–14	2311 (0.3)	572 (0.2)	464 (0.3)	473 (0.4)	802 (0.4)	
≥ 15	1609 (0.2)	770 (0.3)	241 (0.2)	293 (0.2)	305 (0.2)	

BMI, body mass index; Q, Quartile

**Fig. 2:**
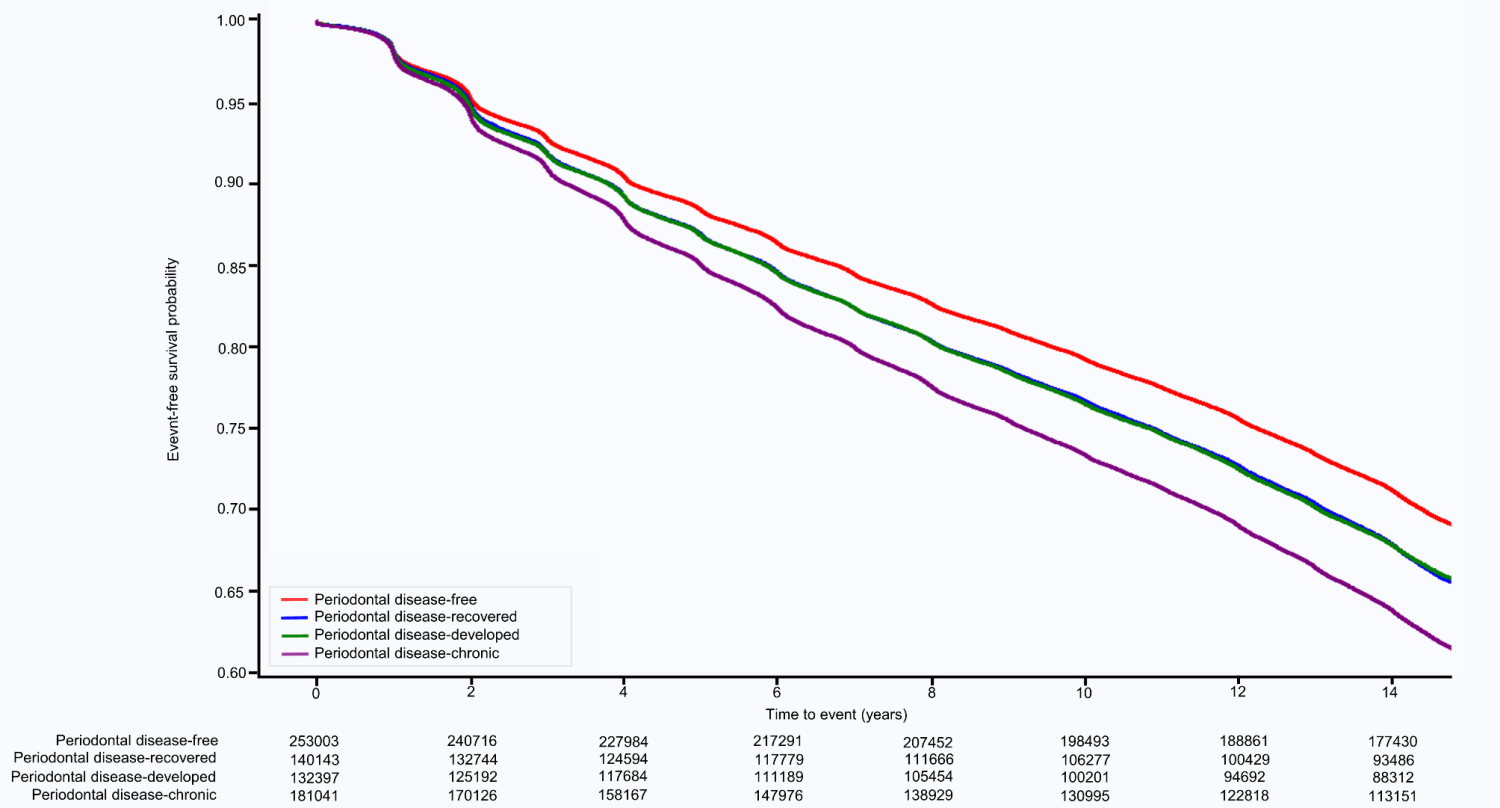
Kaplan-Meier survival curves for hypertension occurrence according to periodontal disease status change

Over a median follow-up duration of 14.3 years, 239,937 (34.0%) cases of hypertension were recorded. Figure 2 shows the Kaplan-Meier survival curves, depicting hypertension occurrences based on changes in PD status. The participants displayed altered hypertension risks linked to PD status changes. Throughout the follow-up period, the PD-chronic group exhibited the highest hypertension risk, whereas the risk remained comparable between the PD-developed and PD-recovered groups. The PD-free group presented the lowest hypertension risk (Table 2). In the multivariate analysis, the PD-recovered group (HR: 1.06, 95% CI: 1.04–1.07, *p* < 0.001), PD-developed group (HR: 1.03, 95% CI: 1.02–1.05, *p* < 0.001), and PD-chronic group (HR: 1.10, 95% CI: 1.09–1.11, *p* < 0.001) displayed elevated hypertension risks compared to the PD-free group (p for trend < 0.001) (Table 2, Supplementary Table 1).

**Table 2:** The association between change in periodontal disease and incident hypertension risk

Group	Event rate (%)	Person-years	Incidence rate (per 1000 person-years)	Unadjusted			Adjusted*		
				HR (95% CI)	*p*-value	*p* for trend	HR (95% CI)	*p*-value	*p* for trend
**Periodontal disease -free**	30.70	3,122,011	24.88	1 (reference)		< 0.001	1 (reference)		< 0.001
**Periodontal disease - recovered**	34.26	1,691,247	28.39	1.14 (1.13, 1.16)	< 0.001		1.06 (1.04, 1.07)	< 0.001	
**Periodontal disease -developed**	34.03	1,594,439	28.26	1.14 (1.12, 1.15)	< 0.001		1.03 (1.02, 1.05)	< 0.001	
**Periodontal disease -chronic**	38.22	2,119,233	32.65	1.32 (1.30, 1.33)	< 0.001		1.10 (1.09, 1.11)	< 0.001	

*The multivariable model was adjusted for age, sex, body mass index, household income, smoking status, alcohol consumption, exercise frequency, presence of comorbidities (diabetes mellitus, dyslipidemia, atrial fibrillation, cancer, and renal disease), Charlson Comorbidity Index, and number of tooth loss

CI, confidence interval; HR, hazard ratio

In additional pairwise analysis (Table 3), the PD-chronic group displayed a comparatively increased hypertension risk in contrast to the PD-recovered group (HR: 1.04, 95% CI: 1.02–1.05, *p* < 0.001). The PD-developed group exhibited a heightened hypertension risk in comparison to the PD-free group (HR: 1.03, 95% CI: 1.02–1.04, *p* < 0.001).

**Table 3:** Pairwise comparisons of the association between change in periodontal disease and incident hypertension risk

	Unadjusted		Adjusted*	
	HR (95% CI)	*p*-value	HR (95% CI)	*p*-value
**Periodontal disease- recovered** **vs. Periodontal disease-free (reference)**	1.14 (1.13, 1.16)	< 0.001	1.03 (1.01, 1.04)	< 0.001
**Periodontal disease-developed** **vs. Periodontal disease-free (reference)**	1.14 (1.12, 1.15)	< 0.001	1.03 (1.02, 1.04)	< 0.001
**Periodontal disease-chronic** **vs. Periodontal disease-free (reference)**	1.32 (1.30, 1.33)	< 0.001	1.07 (1.06, 1.08)	< 0.001
**Periodontal disease-developed** **vs. Periodontal disease- recovered (reference)**	1.00 (0.98, 1.01)	0.544	0.98 (0.97, 1.00)	0.007
**Periodontal disease- chronic** **vs. Periodontal disease- recovered (reference)**	1.15 (1.14, 1.17)	< 0.001	1.04 (1.02, 1.05)	< 0.001
**Periodontal disease- chronic** **vs. Periodontal disease-developed (reference)**	1.16 (1.14, 1.17)	< 0.001	1.07 (1.06, 1.08)	< 0.001

*The multivariable model was adjusted for age, sex, body mass index, household income, smoking status, alcohol consumption, exercise frequency, presence of comorbidities (diabetes mellitus, dyslipidemia, atrial fibrillation, cancer, and renal disease), Charlson Comorbidity Index, and number of tooth loss

CI, confidence interval; HR, hazard ratio

In sensitivity analysis, the association of PD status with risk of hypertension was consistently noted (Supplementary Table 2). Furthermore, the results of pairwise comparison also demonstrated a consistent association between the PD-recovered group and the PD-chronic group. The PD-chronic group showed a higher risk of hypertension compared to that of the PD-recovered group (HR: 1.03, 95% CI: 1.02–1.15, *p* < 0.001) (Supplementary Table 3).

## Discussion

The main results of our study indicate that the occurrence of hypertension is influenced by PD dynamics. Specifically, individuals with persistent PD had the highest risk of developing hypertension, while those who had recovered from PD had a lower risk. Additionally, individuals with newly developed PD had a higher risk of hypertension than those who remained free from PD.

Although the adjusted HR for the association between changes in PD status and hypertension are statistically significant, the values are relatively small. This suggests that the observed risk may largely be due to baseline factors like age, sex, body mass index, smoking status, alcohol consumption, and lifestyle, which were adjusted for in the analysis. These factors are well-known contributors to hypertension and may overshadow the impact of PD. Nonetheless, the consistent association across different PD status changes in our large cohort indicates the significant role that PD management could play in hypertension prevention. While PD is not currently established as a risk factor for hypertension, our findings suggest that it may be worth further investigation. Future research should explore the interaction between PD and other risk factors to better understand their combined impact on hypertension, which could inform more comprehensive prevention strategies.

Multiple previous studies have shown a link between PD and hypertension. A prospective cohort study conducted among university students in Japan examined the association between PD and hypertension, revealing a significant correlation between the two conditions [32]. Other studies that utilized data from a nationally representative sample in Korea discovered that individuals with PD, a high number of tooth loss, and poor oral hygiene practices are more likely to have a higher incidence of hypertension, while dental scaling and frequent toothbrushing are protective factors [33, 34]. Meta-analysis studies have shown that patients with PDs have an increased risk of developing hypertension, but evidence that periodontal therapy reduces blood pressure is lacking [35, 36]. Despite PD being a condition that can be treated and controlled, there is a lack of large-scale studies examining the potential link between recovery from or development of PD and changes in the risk of hypertension. The findings of our study provide additional insight into the association between hypertension and changes in PD status.

Although our study did not identify the exact mechanism that links PD with new-onset hypertension, we propose several interconnected hypotheses. Research suggests that ulceration in periodontal pockets can spread pathogens from the oral cavity into the systemic circulation [37], which can cause direct intrusion of the arterial wall by microorganisms resulting in atherosclerosis and inflammation. This vascular inflammation and atherosclerosis may trigger the development of hypertension [38]. Additionally, high virulence with dysbiosis of an oral biofilm caused by PD can lead to indirect propagation of pro-inflammatory cytokines [39]. These cytokines, including interleukin-6 and tumor necrosis factor-alpha, can increase the inflammatory burden both locally and systemically, and these markers are closely associated with the development of hypertension [40]. Moreover, periodontal pathogens such as *Porphyromonas gingivalis* can invade endothelial cells and contribute to endothelial dysfunction, a key factor in the pathogenesis of hypertension [41]. Chronic inflammation due to PD can also enhance oxidative stress, which further impairs endothelial function and promotes hypertension [42]. Additionally, PD may contribute to the development of insulin resistance and metabolic syndrome, both of which are significant risk factors for hypertension [43].

The results of this study suggest that individuals who have recovered from PD have a reduced risk of developing hypertension. Several controlled human studies have shown that dental treatment or good oral hygiene practices can reduce the presence of periodontal bacteria [44], and professional periodontal treatment can lower serum inflammatory biomarkers [45]. These reductions in oral bacteria and inflammation may explain the decreased risk of hypertension among individuals who have recovered from PD. Furthermore, improving periodontal health can enhance endothelial function and reduce oxidative stress [46], contributing to lower blood pressure. These findings emphasize that PD can be treated and managed and that the risk of hypertension resulting from periodontal inflammation can be modified. Further research focusing on the clinical significance of periodontal treatment is needed to better understand its benefits.

This study has several limitations. First, the definition of hypertension used in this study has certain constraints. The Korea Hypertension Fact Sheet 2023 reported that awareness and treatment rates of hypertension are 74.1% and 70.3%, respectively [16]. Consequently, using operational definitions from claims data alone might be prone to under-reporting bias. To address this, we incorporated both claims data and health examination results to define hypertension. This dual approach aimed to capture more cases of hypertension and reduce the bias associated with under-reporting. However, despite these efforts, some degree of under-reporting bias may still exist, which should be considered when interpreting the results. Second, there may be a potential selection bias due to the inclusion criteria. From the initial NHIS cohort of 2.4 million individuals, we included participants with complete data from oral health exams in 2003 and either 2005 or 2006. After excluding those with incomplete data and a history of hypertension, the final study population was reduced to 706,584 participants. However, these rigorous criteria were necessary to enhance the study’s internal validity and accurately examine the relationship between changes in PD status and the risk of hypertension. Third, the database used was derived from previously conducted examinations, which employed a threshold of probing depth greater than 4 mm to define pathological periodontal pockets. Recommended periodontitis case definitions for epidemiological studies by the American Academy of Periodontology encompass clinical attachment loss and probing depth, while the Community Periodontal Index categorizes shallow pockets as greater than 4 mm and deep pockets as greater than 6 mm. Further research with more detailed periodontitis diagnosis, considering the aforementioned definitions, is required to ascertain the influence of periodontal inflammation on hypertension development. Fourth, there could be potential confounding factors not accounted for in this study that may influence the occurrence of hypertension. Fifth, the study groups were established without adjustments for participants’ history of periodontal treatment. Additional research is necessary to explore the clinical implications of periodontal treatment and to determine if the prevention or recovery from PD can effectively reduce the risk of developing hypertension. Sixth, the study was conducted solely on individuals from the Korean population; the results may not be generalizable to other races and ethnicities. Seventh, due to the limited availability of detailed attachment loss data, we could not evaluate the effects of varying degrees of severity of PD in this study. Eighth, while participating dentists underwent training before conducting oral examinations, it is important to note that the database used in the study lacks comprehensive details regarding the calibration of examiners and the agreements reached both within and between examiners.

Nonetheless, this study possesses strengths. We utilized extensive and representative longitudinal data on a national scale, enabling us to examine the impact of alterations in PD on the development of hypertension. Additionally, our main findings were derived from a comprehensive dataset that included many health screenings and complete outcomes. Although the association between PD and hypertension risk at a single time point is well-established, there was a lack of population-scale evidence regarding the potential impact of changing PD status on the risk of hypertension. In this study, we examined a population-based database, incorporating information from routine health examinations and professional dental diagnoses to clarify the altered risk of hypertension in individuals with varying PD statuses. The results emphasize the importance of regular dental examinations and management of PD as a strategy for reducing hypertension risk.

## Conclusions

In conclusion, our study provides evidence that chronic PD is associated with an increased risk of developing hypertension in the general population. Despite the relatively modest hazard ratios, the consistent association across different PD status changes suggests the potential importance of PD management in hypertension prevention. Recovery from PD may have beneficial effects in reducing hypertension risk, emphasizing the need for regular dental examinations and effective management of PD. Future prospective studies are needed to confirm these findings and explore the mechanisms linking PD and hypertension.

## Electronic supplementary material

Below is the link to the electronic supplementary material.

Supplementary Material 1

Supplementary Material 2

## Data Availability

The data used in this study are available in the National Health Insurance Service (NHIS) database; however, restrictions apply to the public availability of the data used under license for the current study. Requests for access to NHIS data can be made through the National Health Insurance Sharing Service homepage [http://nhiss.nhis.or.kr/bd/ab/bdaba021eng.do]. To access the database, a completed application form, research proposal, and application for approval from the applicable institutional review board should be submitted to the NHIS inquiry committee for research support for review.

## Abbreviations

PD: Periodontal disease
NHIS: National Health Insurance Service
ICD: International Classification of Diseases
SBP: Systolic blood pressure
DBP: Diastolic blood pressure
HR: Hazard ratio
CI: Confidence interval
