# Supplementary material for: Chronic periodontal disease is related with newly developing hypertension: a nationwide cohort study
Source: Clin Hypertens. 2024 Oct 1;30:27. doi: 10.1186/s40885-024-00285-6 (PMC11443627; doi:10.1186/s40885-024-00285-6)
Supplement: Supplementary file 1 — Supplementary Material 1 [file 40885_2024_285_MOESM1_ESM.docx]

**Supplementary Methods**

Individual comorbidities between 2002 and the index date were identified. Diabetes mellitus was defined as satisfying one of these criteria: 1) at least one claim for relevant diagnostic codes (ICD-10 E11–14) with a prescription of an anti-diabetic agent, 2) two or more claims for relevant diagnostic codes (ICD-10 E11–14), 3) fasting serum glucose level ≥ 7.0 mmol/L, or 4) diabetes mellitus self-reported in the questionnaire [1-4]. Dyslipidemia was defined as satisfying one of the following criteria: 1) at least one claim for the relevant diagnostic code (ICD-10 E78) with a prescription of dyslipidemia-related agent, 2) two or more claims for the relevant diagnostic code (ICD-10 E78), or 3) total cholesterol ≥ 240 mg/dL [2, 3]. Atrial fibrillation was defined as two or more claims for the relevant diagnostic code (ICD-10 I48) [5-7]. Cancer was defined as having one hospitalization or at least three outpatient claims for a primary diagnosis code (ICD-10 C00-97) with a Rare Incurable Disease (RID) registration code ‘V027’ or ‘V193-4’. The RID registration was designed to cover only 5% or less of total medical expenses, and because it specifically confirmed how the doctor diagnosed cancer, the accuracy of each cancer diagnosis was very high. When this cancer diagnosis algorithm was applied, the overall diagnostic accuracy for each cancer was over 90% [8]. Renal disease was defined as two or more claims for relevant diagnostic codes (ICD-10 N17-19, I12-13, E082, E102, E112, E132) or an estimated glomerular filtration rate less than 60 mL/min/1.73 m^2^ [7, 9]. For investigating burden of comorbidity, Charlson Comorbidity Index (CCI) was applied. The CCI was a method of categorizing comorbidities of patients based on the ICD-10 diagnoses. Each comorbidity category has an associated weight (from 1 to 6), depending on the risk of dying associated with each one. The CCI has been widely used and validated for various applications in health care research [10, 11].

**References**

1. Song TJ, Kim JW, Kim J. Oral health and changes in lipid profile: A nationwide cohort study. J Clin Periodontol. 2020;47(12):1437-45.

2. Woo HG, Chang Y, Lee JS, Song TJ. Association of Tooth Loss with New-Onset Parkinson's Disease: A Nationwide Population-Based Cohort Study. Parkinsons Dis. 2020;2020:4760512.

3. Chang Y, Woo HG, Lee JS, Song TJ. Better oral hygiene is associated with lower risk of stroke. J Periodontol. 2021;92(1):87-94.

4. Lee K, Lee JS, Kim J, Lee H, Chang Y, Woo HG, et al. Oral health and gastrointestinal cancer: A nationwide cohort study. J Clin Periodontol. 2020;47(7):796-808.

5. Kim J, Kim HJ, Jeon J, Song TJ. Association between oral health and cardiovascular outcomes in patients with hypertension: a nationwide cohort study. J Hypertens. 2022;40(2):374-81.

6. Chang Y, Lee H, Song TJ. Association of gamma-glutamyl transferase variability with risk of venous thrombosis. Sci Rep. 2023;13(1):7402.

7. Park JH, Lee H, Kim JW, Song TJ. Association between periodontal disease status and risk of atrial fibrillation: a nationwide population-based cohort study. BMC Oral Health. 2023;23(1):461.

8. Yang MS, Park M, Back JH, Lee GH, Shin JH, Kim K, et al. Validation of Cancer Diagnosis Based on the National Health Insurance Service Database versus the National Cancer Registry Database in Korea. Cancer Res Treat. 2022;54(2):352-61.

9. Song TJ, Chang Y, Jeon J, Kim J. Oral health and longitudinal changes in fasting glucose levels: A nationwide cohort study. PLoS One. 2021;16(6):e0253769.

10. Charlson ME, Carrozzino D, Guidi J, Patierno C. Charlson Comorbidity Index: A Critical Review of Clinimetric Properties. Psychother Psychosom. 2022;91(1):8-35.

11. D'Hoore W, Bouckaert A, Tilquin C. Practical considerations on the use of the Charlson comorbidity index with administrative data bases. J Clin Epidemiol. 1996;49(12):1429-33.
