# Supplementary material for: Chronic periodontal disease is related with newly developing hypertension: a nationwide cohort study
Source: Clin Hypertens. 2024 Oct 1;30:27. doi: 10.1186/s40885-024-00285-6 (PMC11443627; doi:10.1186/s40885-024-00285-6)
Supplement: Supplementary file 2 — Supplementary Material 2 [file 40885_2024_285_MOESM2_ESM.docx]

**Supplementary table 1**. Results of multivariable analysis for occurrence of hypertension.

|  | ***Adjusted HR (95% CI)** | **p-value** | **p for trend** |
| --- | --- | --- | --- |
| **Age (years)** | 1.04 (1.04, 1.05) | <0.001 | <0.001 |
| **Sex** |  |  | <0.001 |
| Men | 1 (reference) |  |  |
| Women | 0.70 (0.70, 0.71) | <0.001 |  |
| **BMI (kg/m2)** | 1.00 (1.00, 1.00) | <0.001 | <0.001 |
| **Household income** |  |  | <0.001 |
| T1, lowest | 1(reference) |  |  |
| T2 | 0.96 (0.95, 0.97) | <0.001 |  |
| T3 | 0.85 (0.84, 0.87) | <0.001 |  |
| T4, highest | 0.74 (0.73, 0.75) | <0.001 |  |
| **Smoking status** |  |  | <0.001 |
| Never | 1 (reference) |  |  |
| Former | 1.01 (1.00, 1.02) | 0.136 |  |
| Current | 1.06 (1.05, 1.07) | <0.001 |  |
| **Alcohol consumption (days/week)** |  |  | <0.001 |
| <1 | 1 (reference) |  |  |
| 1-3 | 0.96 (0.90, 1.03) | 0.268 |  |
| ≥3 | 1.19 (1.11, 1.27) | <0.001 |  |
| **Regular exercise (days/week)** |  |  | <0.001 |
| <3 | 1 (reference) |  |  |
| ≥3 | 1.03 (1.02, 1.04) | <0.001 |  |
| **Comorbidities** |  |  |  |
| Diabetes mellitus | 1.15 (1.13, 1.16) | <0.001 | <0.001 |
| Dyslipidemia | 1.28 (1.27, 1.30) | <0.001 | <0.001 |
| Atrial fibrillation | 1.76 (1.60, 1.94) | <0.001 | <0.001 |
| Cancer | 0.89 (0.86, 0.92) | <0.001 | <0.001 |
| Renal disease | 1.28 (1.21, 1.36) | <0.001 | <0.001 |
| **Charlson Comorbidity Index** |  |  | <0.001 |
| 0 | 1 (reference) |  |  |
| 1 | 1.04 (1.03, 1.05) | <0.001 |  |
| ≥2 | 1.19 (1.17, 1.20) | <0.001 |  |
| **Number of tooth loss** |  |  |  |
| 0 | 1 (reference) |  | <0.001 |
| 1-7 | 1.04 (1.03, 1.06) | <0.001 |  |
| 8-14 | 1.13 (1.07, 1.19) | <0.001 |  |
| ≥15 | 1.21 (1.14, 1.28) | <0.001 |  |
| **Study group** |  |  | <0.001 |
| Periodontal disease-free | 1 (reference) |  |  |
| Periodontal disease-recovered | 1.06 (1.04, 1.07) | <0.001 |  |
| Periodontal disease-developed | 1.03 (1.02, 1.05) | <0.001 |  |
| Periodontal disease-chronic | 1.10 (1.09, 1.11) | <0.001 |  |

*Multivariable model was adjusted for age, sex, body mass index, household income, smoking, alcohol consumption, regular exercise, diabetes mellitus, dyslipidemia, atrial fibrillation, cancer, renal disease, Charlson Comorbidity Index, and number of tooth loss.

CI, confidence interval; HR, hazard ratio.

**Supplementary table 2**. Results of multivariable analysis for occurrence of hypertension (landmark analysis).

|  | ***Adjusted HR (95% CI)** | **p-value** | **p for trend** |
| --- | --- | --- | --- |
| **Age (years)** | 1.05 (1.05, 1.05) | <0.001 | <0.001 |
| **Sex** |  |  | <0.001 |
| Men | 1 (reference) |  |  |
| Women | 0.76 (0.75, 0.77) | <0.001 |  |
| **BMI (kg/m2)** | 1.00 (1.00, 1.00) | <0.001 | <0.001 |
| **Household income** |  |  | <0.001 |
| T1, lowest | 1 (reference) |  |  |
| T2 | 0.97 (0.95, 0.98) | <0.001 |  |
| T3 | 0.86 (0.84, 0.87) | <0.001 |  |
| T4, highest | 0.74 (0.73, 0.75) | <0.001 |  |
| **Smoking status** |  |  | <0.001 |
| Never | 1 (reference) |  |  |
| Former | 1.01 (1.00, 1.02) | 0.142 |  |
| Current | 1.06 (1.05, 1.07) | <0.001 |  |
| **Alcohol consumption (days/week)** |  |  | <0.001 |
| <1 | 1 (reference) |  |  |
| 1-3 | 0.97 (0.91, 1.04) | 0.345 |  |
| ≥3 | 1.19 (1.11, 1.27) | <0.001 |  |
| **Regular exercise (days/week)** |  |  | 0.001 |
| <3 | 1 (reference) |  |  |
| ≥3 | 1.03 (1.02, 1.04) | <0.001 |  |
| **Comorbidities** |  |  |  |
| Diabetes mellitus | 1.15 (1.13, 1.16) | <0.001 | <0.001 |
| Dyslipidemia | 1.27 (1.25, 1.28) | <0.001 | <0.001 |
| Atrial fibrillation | 1.77 (1.61, 1.95) | <0.001 | <0.001 |
| Cancer | 0.93 (0.90, 0.96) | <0.001 | <0.001 |
| Renal disease | 1.28 (1.21, 1.36) | <0.001 | <0.001 |
| **Charlson Comorbidity Index** |  |  | <0.001 |
| 0 | 1 (reference) |  |  |
| 1 | 1.06 (1.05, 1.06) | <0.001 |  |
| ≥2 | 1.21 (1.19, 1.22) | <0.001 |  |
| **Number of tooth loss** |  |  | <0.001 |
| 0 | 1 (reference) |  |  |
| 1-7 | 1.05 (1.03, 1.06) | <0.001 |  |
| 8-14 | 1.13 (1.07, 1.19) | <0.001 |  |
| ≥15 | 1.22 (1.15, 1.30) | <0.001 |  |
| **Study group** |  |  | <0.001 |
| Periodontal disease-free | 1 (reference) |  |  |
| Periodontal disease-recovered | 1.05 (1.04, 1.07) | <0.001 |  |
| Periodontal disease-developed | 1.03 (1.02, 1.04) | <0.001 |  |
| Periodontal disease-chronic | 1.10 (1.09, 1.11) | <0.001 |  |

*Multivariable model was adjusted for age, sex, body mass index, household income, smoking, alcohol consumption, regular exercise, diabetes mellitus, dyslipidemia, atrial fibrillation, cancer, renal disease, Charlson Comorbidity Index, and number of tooth loss.

CI, confidence interval; HR, hazard ratio.

**Supplementary table 3**. Pairwise comparisons of the association between change in periodontal disease and incident hypertension risk (landmark analysis).

|  | **Unadjusted** | | **Adjusted*** | |
| --- | --- | --- | --- | --- |
|  | **HR (95% CI)** | **p-value** | **HR (95% CI)** | **p-value** |
| **Periodontal disease -recovered**  **vs. Periodontal disease-free (reference)** | 1.14 (1.12, 1.15) | <0.001 | 1.03 (1.02, 1.04) | <0.001 |
| **Periodontal disease-developed**  **vs. Periodontal disease-free (reference)** | 1.13 (1.12, 1.14) | <0.001 | 1.03 (1.02, 1.04) | <0.001 |
| **Periodontal disease-chronic**  **vs. Periodontal disease-free (reference)** | 1.30 (1.29, 1.31) | <0.001 | 1.07 (1.06, 1.08) | <0.001 |
| **Periodontal disease-developed**  **vs. Periodontal disease-recovered (reference)** | 0.99 (0.98, 1.01) | 0.993 | 0.98 (0.97, 0.99) | 0.002 |
| **Periodontal disease- chronic**  **vs. Periodontal disease-recovered (reference)** | 1.14 (1.13, 1.16) | <0.001 | 1.03 (1.02, 1.05) | <0.001 |
| **Periodontal disease- chronic**  **vs. Periodontal disease-developed (reference)** | 1.15 (1.14, 1.17) | <0.001 | 1.07 (1.06, 1.08) | <0.001 |

*Multivariable model was adjusted for age, sex, body mass index, household income, smoking, alcohol consumption, regular exercise, diabetes mellitus, dyslipidemia, atrial fibrillation, cancer, renal disease, Charlson comorbidity index, and number of tooth loss.

CI, confidence interval; HR, hazard ratio.
